# Evaluation of Candidate Genes for Cholinesterase Activity in Farmworkers Exposed to Organophosphorus Pesticides: Association of Single Nucleotide Polymorphisms in *BCHE*

**DOI:** 10.1289/ehp.0901764

**Published:** 2010-06-08

**Authors:** Timothy D. Howard, Fang-Chi Hsu, Joseph G. Grzywacz, Haiying Chen, Sara A. Quandt, Quirina M. Vallejos, Lara E. Whalley, Wei Cui, Stephanie Padilla, Thomas A. Arcury

**Affiliations:** 1 Center for Genomics and Personalized Medicine Research; 2 Department of Biostatistical Sciences, Division of Public Health Sciences; 3 Department of Family and Community Medicine and; 4 Department of Epidemiology and Prevention, Division of Public Health Sciences, Wake Forest University School of Medicine, Winston-Salem, North Carolina, USA; 5 Genetics and Cellular Toxicology Branch, Integrated Systems Toxicology Division, National Health and Environmental Effects Research Laboratory, U.S. Environmental Protection Agency, Research Triangle Park, North Carolina, USA

**Keywords:** BCHE, butyrylcholinesterase, cholinesterase, farmworkers, genetics, organophosphate pesticides, SNPs

## Abstract

**Background:**

Organophosphate pesticides act as cholinesterase inhibitors. For those with agricultural exposure to these chemicals, risk of potential exposure-related health effects may be modified by genetic variability in cholinesterase metabolism. Cholinesterase activity is a useful, indirect measurement of pesticide exposure, especially in high-risk individuals such as farmworkers. To understand fully the links between pesticide exposure and potential human disease, analyses must be able to consider genetic variability in pesticide metabolism.

**Objectives:**

We studied participants in the Community Participatory Approach to Measuring Farmworker Pesticide Exposure (PACE3) study to determine whether cholinesterase levels are associated with single-nucleotide polymorphisms (SNPs) involved in pesticide metabolism.

**Methods:**

Cholinesterase levels were measured from blood samples taken from 287 PACE3 participants at up to four time points during the 2007 growing season. We performed association tests of cholinesterase levels and 256 SNPs in 30 candidate genes potentially involved in pesticide metabolism. A false discovery rate (FDR) *p*-value was used to account for multiple testing.

**Results:**

Thirty-five SNPs were associated (unadjusted *p* < 0.05) based on at least one of the genetic models tested (general, additive, dominant, and recessive). The strongest evidence of association with cholinesterase levels was observed with two SNPs, rs2668207 and rs2048493, in the butyrylcholinesterase (*BCHE*) gene (FDR adjusted *p* = 0.15 for both; unadjusted *p* = 0.00098 and 0.00068, respectively). In participants with at least one minor allele, cholinesterase levels were lower by 4.3–9.5% at all time points, consistent with an effect that is independent of pesticide exposure.

**Conclusions:**

Common genetic variation in the *BCHE* gene may contribute to subtle changes in cholinesterase levels.

Epidemiological studies on the potential effects of organophosphate pesticide exposure have provided conflicting results, especially with chronic, low levels of exposures where immediate effects are absent (reviewed by Ray 1998; Ray and Richards 2001). One potential effect modifier in these studies is genetic variability of pesticide metabolism genes, which may allow some individuals to metabolize pesticides at greater rates than others.

The effective dose of pesticide exposure an individual receives is dependent on the initial environmental exposure, but it is also influenced by an individual’s innate ability to detoxify and excrete specific pesticides or classes of pesticides. This is partially determined by the genes that encode for proteins involved in these metabolic processes. Genetic variation of these genes likely leads to differential ability to metabolize these toxicants, leading to variability in the susceptibility to the effects of chronic exposure. Two protein families that metabolize organophosphorus pesticides are the cytochrome P450s and the paraoxonases (PONs), specifically PON1 (EC 3.1.1.2). Polymorphisms in *PON1* have been reported to be correlated with levels of PON1 enzyme (reviewed by Costa et al. 2003), suggesting that genetic variation contributes to the level or activity of this enzyme. A comprehensive analysis of the genes potentially involved in organophosphorus pesticide metabolism is necessary to identify the individual single-nucleotide polymorphisms (SNPs) that contribute to individual variability in organophosphorus pesticide metabolism.

Organophosphates are potent cholinesterase inhibitors (Costa 2006). We previously reported that the number of organophosphorus and carbamate pesticides detected in the urine of farmworkers predicted reductions in cholinesterase activity, independent of other confounders (Quandt et al. 2010). Therefore, cholinesterase activity can provide an indirect measurement of pesticide exposure (reviewed by Nigg and Knaak 2000). The goal of this study was to characterize genetic variation of specific genes involved in organophosphorus pesticide metabolism in an effort to identify those individuals most at risk of the potential adverse health effects from chronic organophosphate exposure.

## Material and Methods

### Population

The Community Participatory Approach to Measuring Farmworker Pesticide Exposure (PACE3) study builds on > 10 years of community-based participatory research conducted by a partnership of Wake Forest University School of Medicine and the North Carolina Farmworkers Project. PACE3 used a two-stage procedure to select farmworkers; details are presented elsewhere (Arcury et al. 2009a; Quandt et al. 2010). Briefly, three partnering agencies prepared lists of farmworker camps for the counties that they served. Camps were randomized and approached in order until each agency recruited a minimum number of camps and a specified number of participants. In total, 287 farmworkers were recruited. Of all farmworkers approached by the interviewers, 13 chose not to participate, for a participation rate of 95.7%. At the second round of data collection, 41 participants were lost to follow-up; 20 were lost at the third round, and 12 were lost at the fourth round. Four rounds of data collection were completed with 197 farmworkers, three rounds with 27, two rounds with 14, and one round with 49.

Community partners for this project included the North Carolina Farmworkers Project (Benson, NC), Green County Health Care, Inc. (Snow Hill, NC), and Columbus County Community Health Center, Inc. (Whitesville, NC). PACE3 used a repeated measures design in which data were collected from participants up to four times at monthly intervals. All sampling, recruitment, data collection, and genetic protocols were approved by the Wake Forest University School of Medicine Institutional Review Board. All participants provided written informed consent prior to the study.

### Cholinesterase measurement

Blood samples to measure cholinesterase activity were collected at each interview (up to four total). Farmworkers cleaned their fingers with alcohol wipes, and data collectors pricked a finger of each farmworker using a sterile lancet. The resulting blood drops were applied to 903 Protein Saver paper (Whatman Ltd., Piscataway, NJ), soaking through a printed half-inch circle that holds 75–80 μL blood. Samples were labeled, allowed to dry, placed in a paper envelope, and then sealed in a plastic bag for transport to the laboratory.

The dried blood samples were delivered to the U.S. Environmental Protection Agency (Cellular and Molecular Toxicology Branch, Neurotoxicology Division, National Health and Environmental Effects Research Laboratory) and analyzed using the method of Hilborn and Padilla (2004). Briefly, dried blood spots were punched out of the filter paper with a standard hole punch. Only those that were 98–100% saturated with blood on both sides of the filter paper were used for the final analysis (90.2% of the samples met these criteria). Previous work had estimated that each punch contained approximately 15 μL fresh blood (Hilborn and Padilla 2004). The punch was then added to 500 μL Triton/Ellman buffer (0.1 mM sodium phosphate buffer, pH 8.0, plus 1% Triton X-100). To allow the whole blood to elute, each vial containing buffer and the punch was refrigerated for 17 hr and then placed in a shaking water bath (26°C) for an additional 4 hr. The Triton/Ellman buffer was then removed from the vial and analyzed for cholinesterase activity using 15 μL of each sample and a basic Ellman assay (Ellman et al. 1961) modified for use in a microtiter plate reader (Nostrandt et al. 1993). All samples from a participant were analyzed on the same day. A reference sample (rat brain homogenate) was run on each plate to ensure consistency among plates; the coefficient of variation for these reference samples was 0.05. Activity was reported as nanomoles acetylthiocholine hydrolyzed per minute per milliliter of whole blood.

### SNP selection and genotyping

During the first data collection visit, participants provided saliva for DNA isolation using the OraGene DNA Self-Collection Kit (DNA Genotek, Inc. Kanata, Ontario, Canada), and DNA was isolated according to the manufacturer’s instructions. We used a strategy that allowed for the maximum genetic information, in addition to potential functional SNPs from each locus, to select SNPs from genomic regions containing genes hypothesized to be involved with pesticide metabolism. Where several genes were clustered in a chromosomal region, the entire genomic region was tested (e.g., *PON1*, *PON2*, and *PON3*). We thoroughly evaluated each locus using the Tagger program (de Bakker et al. 2005) to identify tagging SNPs based on both the Centre d’Etude du Polymorphisme Humain (CEPH) Utah (CEU) and combined Asian [Han Chinese in Beijing, China (CHB), plus Japanese in Tokyo, Japan (JPT)] populations from the International HapMap Project (International HapMap Consortium 2003). The pairwise tagging algorithm was used with an *r*^2^ of 0.80 in the CEU and CHB + JPT populations separately, and then redundant SNPs were removed. Potential functional SNPs [those that were located in coding regions or were evolutionarily conserved based on the conservation track of the UCSC Genome Browser (University of California, Santa Cruz; http://genome.ucsc.edu) were also given priority. Thirty-four previously described ancestry informative markers (AIMs) were included so that genetic ancestry could be evaluated and used as a covariate for all statistical analyses (Choudhry et al. 2006). Genotyping was performed using the GoldenGate assay and the BeadArray 500GX reader (Illumina Inc., San Diego, CA). Clustering of alleles and initial quality control steps were performed with BeadStudio (Illumina Inc.). We selected a total of 336 SNPs from the candidate loci based on these criteria; of these, 287 were genotyped successfully and analyzed. From the entire population, 265 samples were genotyped successfully with call rates of 94.5–100%. Most of the samples (*n* = 256) had call rates ≥ 99%.

### Statistical analyses

Tests for Hardy-Weinberg equilibrium (HWE) were performed for each SNP using an exact test (Wigginton et al. 2005) implemented in the computer program Haploview (Barrett et al. 2005). We used Structure software (Pritchard et al. 2000) to infer population structure by evaluating the 34 AIMs genotyped in this study. A model with two populations (*k* = 2) showed the best fit (with the lowest natural log likelihood). The proportion of ancestry in population 1 was used as a covariate in the following association analysis. Principal component analysis was also performed separately using JMP Genomics (SAS Institute Inc., Cary, NC) to view the genetic ancestry compared with samples from the International HapMap Project (International HapMap Consortium 2003). We used the three principal components that described the largest proportion of the variance (23.3%, 9.8%, and 5.0%) to generate these data.

We checked cholinesterase values for normality in order to confirm the distributional assumptions of the test and minimize heterogeneity of variance. These values were normally distributed and did not require further transformation for analysis. We evaluated associations between cholinesterase levels measured over the four time points, and genotyped SNPs using a mixed effect model, where random intercept was applied with camp site as the clustering variable; the covariance matrix was estimated at the individual within-camp level. A general test of association (the two degree-of-freedom test of genotypic association) and three individual contrasts defined by *a priori* genetic models (additive, dominant, and recessive) were computed. Associations were adjusted for age, sex, proportion of ancestry, and time period. We used false discovery rate (FDR) (Benjamini and Hochberg 1995; Benjamini and Yekateuli 2001) to adjust for the multiple comparisons. FDR controls the expected proportion of falsely rejected hypotheses (the FDR). Its threshold is determined from the observed *p*-value distribution and is adaptive to the amount of significant results in the data; the FDR is less conservative than the commonly used Bonferroni adjustment. In this study, we calculated FDR-adjusted *p*-values using the procedure “multtest” in SAS (SAS Institute Inc.)

## Results

The data used in this analysis were collected from 287 individuals in 2007 as part of a community-based participatory research project conducted in east central North Carolina (Table 1). We successfully genotyped 291 SNPs from 16 chromosomal regions containing candidate genes for metabolism of organophosphate pesticides (Table 2). These regions encompassed 30 individual candidate genes, including P450 genes involved in metabolism of organophosphates (*CYP1B1*, *CYP3A4*, *CYP3A5*, *CYP3A7*, *CYP3A43*, *CYP2C8*, *CYP2C9*, *CYP2C18*, *CYP2C19*, *CYP2E1*, *CYP1A1*, *CYP1A2*, and *CYP2B6*), the genes that encode for acetylcholinesterase (*ACHE*) and butyrylcholinesterase (*BCHE*), and the *PON* gene cluster (*PON1*, *PON2*, and *PON3*). In this population, 7 SNPs were monomorphic and 28 were not consistent with the HWE (*p* < 0.01), so we did not consider analysis with these SNPs. In total, we considered 256 SNPs appropriate for analysis.

**Figure 1:**
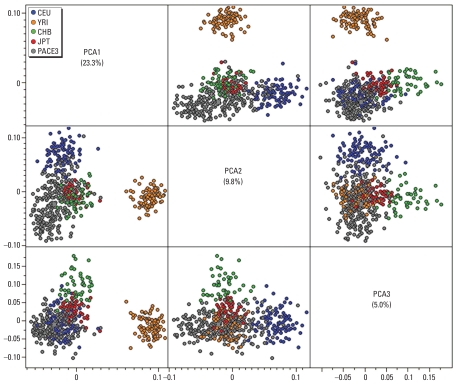
Genetic ancestry of PACE3 participants based on principal component analysis (PCA) with International HapMap samples. Three principal components (PCA1–PCA3) are shown that discriminate the HapMap samples from Northern European (CEU), Yoruban in Ibadan, Nigeria (YRI), Chinese (CHB), and Japanese (JPT) genetic ancestry. Each panel is a plot of two principal component values. PACE3 individuals cluster between the CEU and Asian populations.

Most of the individuals in PACE3 reported being of Mexican descent. Principal component analysis with the 34 AIMs was consistent with this designation, because most of the of individuals clustered between the HapMap samples for individuals of Northern European (CEU) and Asian (CHB and JPT) genetic ancestry (Figure 1). Principal component 1 clearly distinguished the Yoruban population, whereas principal component 2 provided the best separation between the Northern European and Asian populations.

**Figure 2:**
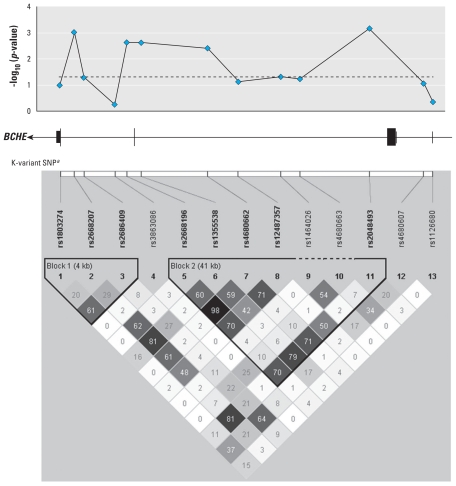
Association data for the *BCHE* gene. (Top) The association of *BCHE* with cholinesterase activity (dominant model). (Middle) The genomic structure of *BCHE*, with exons indicated by vertical lines and boxes. (Bottom) The linkage disequilibrium LD pattern (*r*^2^ values are provided). ^a^The K-variant (rs1803274), reported previously by Rubinstein et al. (1978), is at the 3′ end of the gene. Bold SNPs are located within haplotype blocks and have a minor allele frequency ≥ 0.05.

**Figure 3:**
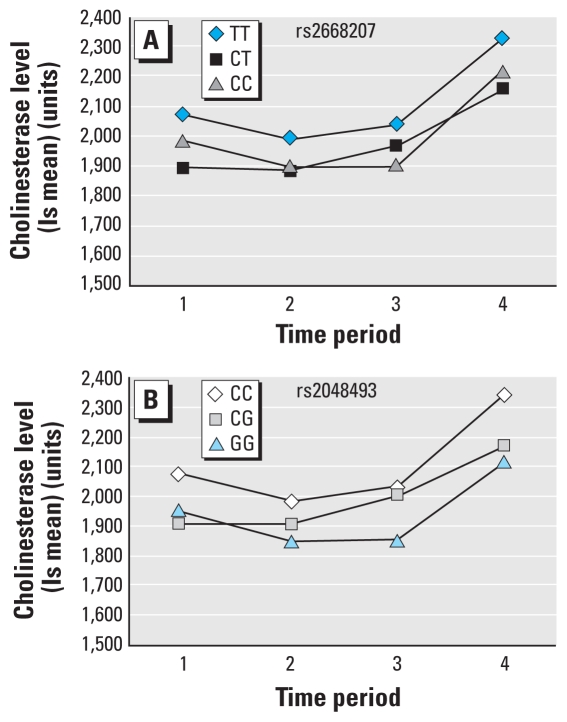
Cholinesterase activity of individuals with rs2668207 (*A*) and rs2048493 (*B*) genotypes. ls, least-squares mean. The four time periods when cholinesterase levels were collected were 1 May to 8 June 2007 (1); 9 June to 7 July 2007 (2); 8 July to 5 August 2007 (3); and 6 August to 4 September 2007 (4).

Twenty-six SNPs were significant at the 0.05 level (unadjusted *p*-values) in at least one of the models tested [general, additive, dominant, and recessive; see Supplemental Material, Table 1 (doi:10.1289/ehp.0901764)]. The top five SNPs associated with the general test of association were located in the *BCHE* gene, and the dominant model seemed most appropriate based on the phenotype and genotype data (Table 3, Figure 2). We observed the strongest evidence of association with two SNPs, rs2668207 and rs2048493 (Table 3; unadjusted *p* = 0.00098 and 0.00068, respectively; FDR adjusted *p* = 0.15 for both SNPs), which were in strong linkage disequilibrium (LD; *r*^2^ = 0.81). A dominant effect was consistent with the pattern of cholinesterase levels over time, where individuals carrying one or more minor alleles had lower cholinesterase activity than did individuals homozygous for the major allele, even over multiple time points (Figure 3). The results for all SNPs analyzed are provided in Supplemental Material, Table 1 (doi:10.1289/ehp.0901764).

## Discussion

Organophosphorus pesticides act as cholinesterase inhibitors and thus may lead to an increase in the neurotransmitter acetylcholine. We have evaluated 287 SNPs in a set of 30 genes potentially involved in the metabolism or detoxification of organophosphate pesticides in a unique farmworker population. SNPs with the strongest evidence of association with cholinesterase activity were located in the *BCHE* gene. For the two SNPs with the highest levels of significance in *BCHE*, individuals with the major allele genotype had the highest cholinesterase activity over all four time periods. Individuals who were heterozygous or homozygous for the minor allele had significantly lower cholinesterase activity.

Mutations within *BCHE* have been associated previously with altered BCHE activity (Rubinstein et al. 1978), many of which cause nonsynonymous coding changes. Sixteen variants—described in the Online Mendelian Inheritance in Man (OMIM) database (http://www.ncbi.nlm.nih.gov/omim)—tend to be strongly associated with BCHE activity. Many of these variants are rare and have low homozygous frequencies (e.g., 1/150,000) or have been identified in isolated families. One of the more common variants of *BCHE*, the well-studied “K” variant (Ala539Thr; rs1126680), is estimated to have a homozygous frequency of 1% and has been shown to decrease the activity of BCHE by 33% (Rubinstein et al. 1978). These data are consistent with genetic variability and human complex phenotypes, for which rare, deleterious mutations (e.g., deletions, nonsynonymous amino acid substitutions) have a strong impact on the function of a protein, and common, conservative polymorphisms (e.g., intronic, synonymous amino acid changes) lead to more subtle changes. This is the first reported analysis of common genetic variation in *BCHE* with cholinesterase activity. As opposed to the rare mutations, the present data suggest that common *BCHE* variations, particularly rs2668207 and rs2048493, are associated with subtle changes (4.3–9.5%) in cholinesterase activity across all four time points. Although this result is not surprising, these decreases could also be due to a marked decrease in activity of either BCHE or ACHE, which we measured simultaneously in our assay. For example, if variation in BCHE is controlled by the *BCHE* gene but variation of ACHE is not, then our ability to identify this association may be masked when both are measured simultaneously. The overall change in cholinesterase activity throughout the growing season may be due to the timing of pesticide application because of the various crops being grown (Arcury et al. 2009b; Quandt et al. 2010).

The overall pattern of cholinesterase activity was similar between genotypes over the four time periods, and individuals with the risk genotypes had the lowest cholinesterase activity, especially at the second and third time points. These decreases are relatively minor, and it remains to be determined if these subtle changes, over time, increase the risk of chronic diseases associated with cholinesterase inhibition.

Surprisingly, we did not observe associations with any SNPs in *PON1*, an esterase associated with high-density lipoproteins that detoxifies some organophosphate pesticides and thus prevents the pesticides from inhibiting cholinesterase. Polymorphisms in *PON1* have been associated with PON1 concentration (Holland et al. 2006) or activity (Furlong et al. 2006; Holland et al. 2006). Both promoter and coding SNPs have been examined, as well as haplotype analyses combining the two groups (Holland et al. 2006). These studies strongly suggest that genetic variation in *PON1* partially regulates the ability of PON1 to detoxify organophosphorus pesticides. To comprehensively evaluate *PON1* and the two nearby genes, *PON2* and *PON3*, we genotyped 57 SNPs, encompassing > 136 kb on chromosome 7q. These SNPs included rs662 (Q192R) and rs854560 (L55V), which have been examined previously in multiple studies (Furlong et al. 2006; Holland et al. 2006). These studies have identified associations predominately with levels of serum cholinesterase, which is predominately BCHE (Hofmann et al. 2009; Sirivarasai et al. 2007). Given that our assay measured a combination of BCHE and ACHE, the effect of *PON1* genetic variation on BCHE may have been dampened. In addition, the Mexican ancestry of our population may have contributed to the lack of association with *PON1* SNPs. Although the previously studied SNPs are believed to be functional, they could be in LD with other causal SNPs that were not captured by the underlying LD structure in our population.

As with any genetic association study, several limitations must be addressed. First, we evaluated 287 SNPs in 30 candidate genes, so there is an increased risk of identifying false positives due to multiple testing. A simple Bonferroni adjustment based on 287 tests results in a *p*-value of 0.000174. Our top SNPs approached this level of significance (for rs2048493, *p* = 0.00068), but they did not meet this threshold. Additional analysis using the FDR approach to account for multiple testing resulted in *p*-values of 0.15 for both of our top SNPs in *BCHE*. Although these results are not statistically significant (i.e., *p* > 0.05), they are interesting and warrant further studies in additional comparable populations. Second, our sample size, although large for a study of migrant and seasonal farmworkers, is small for a genetic association study. This is alleviated somewhat by the longitudinal measurement of cholinesterase activity over four time points and the consistency of the results by genotype of the SNPs in *BCHE*. Finally, it is possible that the SNPs in *BCHE* that are associated with cholinesterase activity are not the actual causative SNPs; instead, other nearby SNPs in LD—or a combination of SNPs (e.g., a haplotype)—are likely to be responsible. Further studies in additional populations are required to determine the true genetic contributors in this genomic region.

Investigators have begun to examine the link between pesticide exposure, particularly exposures to organophosphorus pesticides, and genetic variation among farmworkers and other agricultural workers (Furlong et al. 2005, 2006; Holland et al. 2006). However, a comprehensive analysis of the genetic variation, combined with a thorough collection of environmental and behavioral data in farmworkers exposed to pesticides, has not been performed to date. We have evaluated 287 SNPs in 30 genes potentially involved in organophosphorus pesticide metabolism or excretion. Our results indicate that SNPs in *BCHE* may contribute to the activity of cholinesterase, a target of organophosphorus pesticide. Farmworkers with the risk genotype had the lowest levels of cholinesterase activity. Additional studies of this gene are required to confirm these findings and to identify potential mechanisms of action.

## Figures and Tables

**Table 1:** Personal characteristics of farmworkers, eastern North Carolina, 2007 (*n* = 287).

Characteristic	*n* (%)
Sex	
Male	262 (91.3)
Female	25 (8.7)

Age (years)	
18–24	63 (22.0)
25–29	55 (19.2)
30–39	92 (32.1)
≥ 40	77 (26.8)

Educational attainment (years)	
0–6	149 (51.9)
7–9	97 (33.8)
≥ 10	41 (14.3)

Country of birth	
Mexico	272 (94.8)
United States	3 (1.0)
Other	12 (4.2)

Country of residence	
Mexico	244 (85.0)
United States	32 (11.1)
Other	11 (3.8)

Language	
English	33 (11.5)
Spanish	287 (100)
Indigenous language	67 (23.3)

Seasons in U.S. agriculture (years)	
≤ 1	48 (16.7)
2–3	49 (17.1)
4–7	82 (28.6)
≥ 8	107 (37.3)

Worker type	
Migrant	255 (88.8)
Seasonal	32 (11.1)

H2A visa	
No	137 (47.7)
Yes	150 (52.3)

Housing	
Grower provided	260 (90.6)
Other	27 (9.4)

Housing type	
House	117 (40.8)
Barracks	58 (20.2)
Trailer	112 (39.0)

**Table 2:** Candidate genes tested for association with cholinesterase activity.

Chromosome	Position (NCBI Build 36)	Region size (kb)	Genes in region	No. SNPs genotyped
1p13	chr1:110032012–110037970	6	*GSTM1*	1
1q24	chr1:169317930–169580099	26	*FMO1*, *FMO2*, *FMO3*, *FMO4*	77
2p22	chr2:38147818–38157803	10	*CYP1B1*	3
3p21	chr3:49686778–49696389	10	*APEH*	3
3q26	chr3:166973387–167037944	65	*BCHE*	13
7q21	chr7:94764502–94903130	160	*PON1*, *PON2*, *PON3*	57
7q22	chr7:99028551–99308888	280	*CYP3A4*, *CYP3A5*, *CYP3A7*, *CYP3A43*	19
7q22	chr7:100325448–100332411	7	*ACHE*	10
10q23	chr10:96392874–96828191	436	*CYP2C8*, *CYP2C9*, *CYP2C18*, *CYP2C19*	49
10q26	chr10:135189996–135204549	15	*CYP2E1*	9
17q13	chr11:67107761–67110712	3	*GSTP1*	8
15q24	chr15:72795630–72838597	43	*CYP1A1*, *CYP1A2*	11
16p11	chr16:28503439–28546981	44	*SULT1A1*, *SULT1A2*	6
16q12	chr16:54392027–54429859	38	*CES1*	4
16q22	chr16:65523286–65566894	44	*CES2*, *CES3*	6
19q13	chr19:46174140–46222459	50	*CYP2B6*	11

Abbreviations: APEH, *N*-acylaminoacyl-peptide hydrolase; CES, carboxylesterase; FMO, flavin containing monooxygenase; GSTM, glutathione *S*-transferase mu; GSTP, glutathione *S*-transferase pi; NCBI, National Center for Biotechnology Information (http://www.ncbi.nlm.nih.gov/); SULT, sulfotransferase.

**Table 3:** Association of SNPs in *BCHE* with cholinesterase activity.

SNP	Position^a^	HWE *p*-value	MAF	Unadjusted *p*-values				FDR *p*-values (dominant)
				General	Additive	Dominant	Recessive	
rs1803274	166,973,974	0.59	0.1	0.24	0.092	0.10	0.48	—
rs2668207^b^	166,976,418	0.4	0.34	0.0044	0.0048	0.00098	0.51	0.15
rs2686409	166,978,094	0.5	0.14	0.032	0.016	0.052	0.025	—
rs3863086	166,983,379	1	0.013	ND	0.55	0.55	ND	—
rs2668196	166,985,403	0.78	0.27	0.0073	0.016	0.0023	0.93	0.18
rs1355538	166,987,871	0.21	0.38	0.0088	0.017	0.0023	0.53	0.18
rs4680662	166,999,244	0.65	0.27	0.012	0.022	0.0039	0.93	0.24
rs12487357	167,004,510	0.67	0.21	0.17	0.15	0.075	0.88	—
rs4680663	167,015,102	1	0.036	ND	0.058	0.058	ND	—
rs2048493^b^	167,026,996	0.5	0.34	0.0026	0.0011	0.00068	0.12	0.15
rs4680607	167,036,281	0.9	0.1	0.021	0.027	0.088	0.011	—
rs1126680	167,037,819	1	0.015	ND	0.45	0.45	ND	—

Abbreviations: MAF, minor allele frequency; NCBI, National Center for Biotechnology Information; ND, cell values were too small for *p*-values to be determined.

a Nucleotide position is based on chromosome 3, NCBI Build 36 (http://www.ncbi.nlm.nih.gov/).

b SNPs with strongest observed association.
